# Coronary intravascular imaging: a piece of history

**DOI:** 10.3389/fcvm.2025.1632513

**Published:** 2025-09-30

**Authors:** Waiel Abusnina, Hector M. Garcia-Garcia, Pablo M. Rubio, Gary S. Mintz, Ron Waksman

**Affiliations:** Section of Interventional Cardiology, MedStar Washington Hospital Center, Washington, DC, United States

**Keywords:** IVUS (intravascular ultrasound), optical coherence tomography, NIRS (near infrared spectroscopy), intravascular imaging, coronary artery disease

## Abstract

Since its early exploration in the 1950s, intracoronary imaging, including intravascular ultrasound (IVUS), optical coherence tomography (OCT), and near-infrared spectroscopy (NIRS), has revolutionized our understanding of coronary artery disease and improved percutaneous coronary intervention outcomes. These technologies have continuously evolved, enhancing our ability to diagnose and treat CAD and ultimately leading to better patient outcomes. This review focuses on the history and key developments of IVUS, OCT, and NIRS.

## Introduction

Unarguably, coronary imaging has been one of the most transformative tools on the investigation of coronary artery disease (CAD). From its inception, intracoronary imaging, with the use of miniature catheters, has continuously shed light on our understanding of the atherosclerosis disease.

In this new era of DESs, not only simple lesions but also more complex lesions were treated by PCI; in this setting, intravascular imaging has played an important role as a clinical support tool for planning and assessing the final results of PCI (1). Intracoronary imaging such as intravascular ultrasound (IVUS) and optical coherence tomography (OCT) have been evaluated in clinical trials for more than two decades and they are now recommended in USA and European guidelines (Table 1). This review offers a journey through the history of the intracoronary imaging, especially developments of IVUS and OCT (Central Illustration).

**Table 1:** American and European guidelines recommendation for the use of intravascular ultrasound (IVUS) and optical coherence tomography (OCT).

Recommendations	Class	Level
2024 ESC/EACTS guidelines on myocardial revascularization		
Intracoronary imaging guidance by IVUS or OCT is recommended for performing PCI on anatomically complex lesions, in particular left main stem, true bifurcations and long lesions.	I	A
When ICA is indicated, IVUS should be considered to evaluate the severity of intermediate stenoses of left main stem prior to revascularization.	IIa	B
2025 ACC/AHA/SCAI guideline for coronary artery revascularization		
In patients with ACS undergoing coronary stent implantation in left main artery or in complex lesions, intracoronary imaging with intravascular ultrasound (IVUS) or optical coherence tomography (OCT) is recommended for procedural guidance to reduce ischemic events	I	A
2021 ACC/AHA/SCAI guideline for coronary artery revascularization		
In patients undergoing coronary stent implantation, IVUS can be useful for procedural guidance, particularly in cases of left main or complex coronary artery stenting, to reduce ischemic events	IIa	B
In patients undergoing coronary stent implantation, OCT is a reasonable alternative to IVUS for procedural guidance, except in ostial left main disease	IIa	B
In patients with stent failure, IVUS or OCT is reasonable to determine the mechanism of stent failure	IIa	C
2018 ESC/EACTS guidelines on myocardial revascularization		
IVUS or OCT should be considered in selected patients to optimize stent implantation	IIa	B
IVUS should be considered to optimize treatment of unprotected left main lesions	IIa	B
IVUS should be considered to assess the severity of unprotected left main lesions	IIa	B
IVUS and/or OCT should be considered to detect stent-related mechanical problems leading to restenosis	IIa	C
2014 ESC/EACTS guidelines on myocardial revascularization		
IVUS in selected patients to optimize stent implantation	IIa	B
IVUS to assess severity and optimize treatment of unprotected left main lesions	IIa	B
IVUS or OCT to assess mechanism of stent failure	IIa	C
OCT in selected patients to optimize stent implantation	IIb	C
2011 ACC/AHA/SCAI guideline for percutaneous coronary intervention		
IVUS is reasonable for the assessment of angiographically indeterminant left main CAD	IIa	B
IVUS and coronary angiography are reasonable 4 to 6 weeks and 1 year after cardiac transplantation to exclude donor CAD, detect rapidly progressive cardiac allograft vasculopathy, and provide prognostic information	IIa	B
IVUS is reasonable to determine the mechanism of stent restenosis	IIa	C
IVUS may be reasonable for the assessment of non–left main coronary arteries with angiographically intermediate coronary stenoses (50% to 70% diameter stenosis)	IIb	B
IVUS may be considered for guidance of coronary stent implantation, particularly in cases of left main coronary artery stenting	IIb	B
IVUS may be reasonable to determine the mechanism of stent thrombosis	IIb	C
IVUS for routine lesion assessment is not recommended when revascularization with PCI or CABG is not being contemplated	III	C
2006 ACC/AHA/SCAI guideline update for percutaneous coronary intervention		
IVUS may be considered for determination of the extent of atherosclerosis in patients with characteristic anginal symptoms and a positive functional study with no focal stenoses or mild CAD on angiography	IIa	C
IVUS may be considered for periinterventional assessment of lesional characteristics and vessel dimensions as a means to select an optimal revascularization device	IIa	C
IVUS may be considered for diagnosis of coronary disease after cardiac transplantation.	IIa	C
IVUS is not recommended when the angiographic diagnosis is clear, and no interventional treatment is planned	III	C

**CENTRAL ILLUSTRATION:**
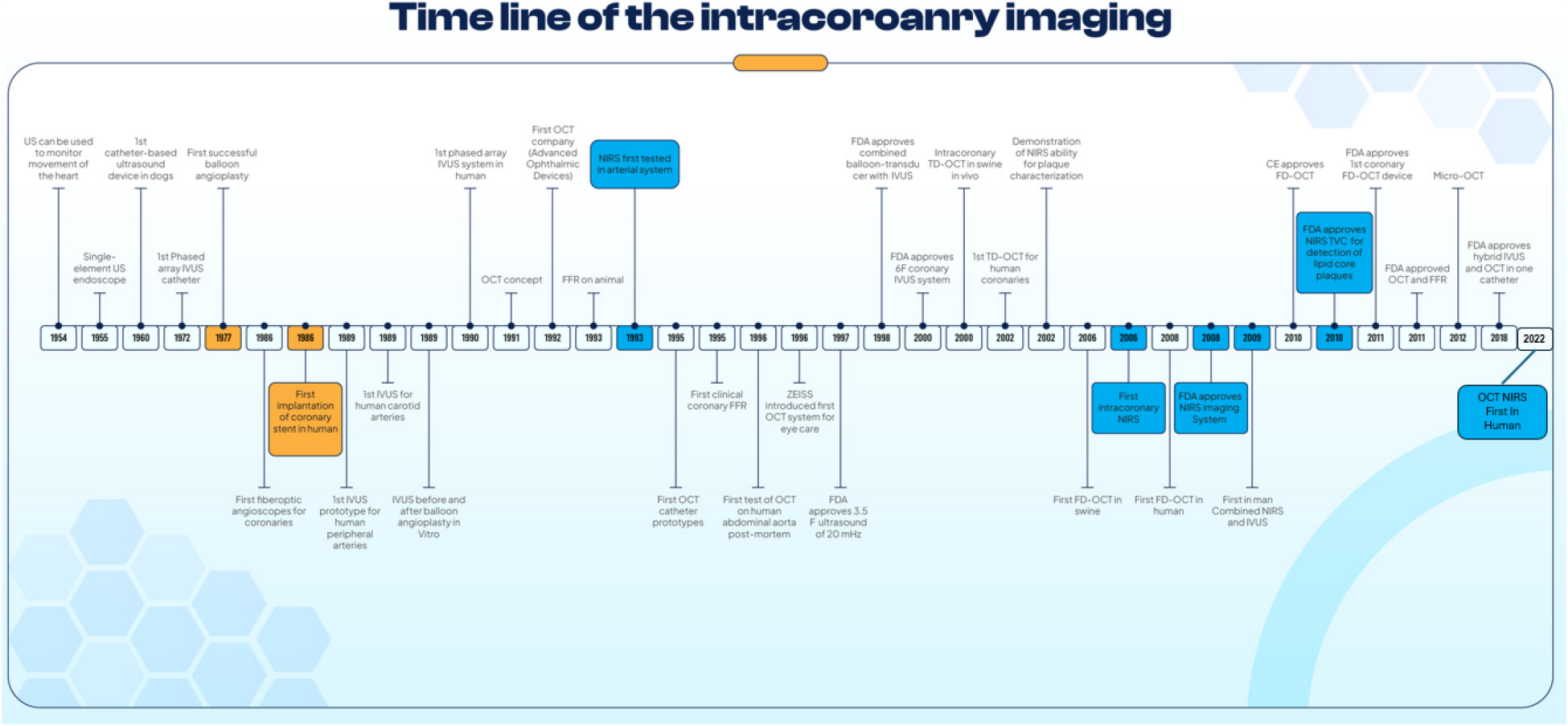
Timeline of Intracoronary Imaging and other Invasive Cardiac Procedures. A figure shows the timeline of the development and key milestones in intracoronary imaging.

### Intravascular ultrasound

It is fascinating to realize that the exploration of heart structure imaging with ultrasound catheter-based devices began in 1954, just two years after Dr. Karl Theodore Dussik presented his groundbreaking paper in 1952 on the use of ultrasound for medical purposes, specifically for examining brain tumors (2). Inge Edler and Carl Hertz introduced the concept of using ultrasound to visualize the internal cardiac structures in 1954. They showed that ultrasound technology could be applied not only for static imaging, but also for monitoring the real-time movement of the heart, marking a significant advancement in medical imaging technology (3). The concept of a probe-mounted ultrasound device dates to 1959. In that year, Dean Franklin's group at the University of Washington in Seattle described an invasive ultrasound flowmeter, but it was clamped around the aorta rather than being intravascular and it was also assessed in animal experiments (4). Subsequently, a polish physician Tomasz Cieszynski, working in Wrocław, Poland, built a small ultrasonic catheter with the goal to visualize the chambers of the canine heart (5). Following these developments, an Australian scientist George Kossoff created an 8 MHz ultrasound transducer with 2 mm diameter mounter on an 8F Cournand catheter in 1966 (6). In the early 1970s, nearly a decade after the introduction of ultrasonic catheters, Nicolaas Bom and his colleagues in Rotterdam, Netherlands, made a significant contribution by developing the first intravascular phased-array catheter. This catheter used a 32-element ultrasound transducer to evaluate human heart chambers and other internal cardiac structures (7).

After the initial enthusiasm, interest in IVUS experienced a period of stagnation and was limited to internally assessing only the heart chambers. However, the scenario changed in the mid-1980s, sparked by several key developments in cardiology, including the development of the first successful percutaneous coronary interventions (8), the first implantation of coronary stent in human (8), and a growing recognition of the limitations of x-ray imaging to provide detailed information about the lumen. For that reason, IVUS was developed further. In the early 1980s, some of the first depictions of human coronary anatomy using ultrasound were produced by Sahn et al. at the University of Arizona (9). They used 9- and 12 MHz surface ultrasound probes to scan epicardial coronary arteries from outside in patients just before undergoing coronary artery bypass grafting. Shortly after, in 1986, C. Todd Sherman at Cedars-Sinai Medical Center made the first attempt to investigate coronary vessels from the inside using flexible fiberoptic angioscopes (10).

However, it wasn't until the late 1980s and early 1990s that IVUS prototypes were more extensively developed and tested. A key figure in this phase was Paul Yock, associated with the University of California, San Francisco, and Stanford University. He developed and introduced one of the first non-commercial IVUS systems (11). Often referred to as the father of IVUS, Yock's system used a 20 MHz transducer and provided some of the earliest *in vivo* images of generic arterial structure. His observations notably included the three-layered appearance of muscular arteries and the early thickening of the tunica intima in atheroma formation. This phenomenon, where a dark ultrasound image appears “behind” a lesion due to its high reflective properties, was a significant discovery (12). In 1989, Jonathan Tobis and John Mallery developed a similar single 20 MHz catheter system (13). This system was tested on various vascular systems, including the coronary, iliac, femoral, and tibial arteries, both before and after balloon dilation angioplasty, with evaluations conducted post-mortem. These efforts of early applications of IVUS generated some of the initial ultrasound images of arterial atherosclerotic lesions, introducing the concept of ultrasound shadowing.

Subsequently, in the early 1990s, John Hodgson and his team finalized their results on one of the first successful “phased array” IVUS trials conducted on conscious patients (14). Although their system also used 20 MHz frequency transducers (Endosonics, Rancho Cordova, CA), their catheter was slightly larger and provided a simultaneous 360° field of view Figure, in contrast to the mechanically rotated side view of earlier models. This groundbreaking phased array model was the first of its kind to be approved for intracoronary imaging in the USA, setting a standard for decades. In 1992, Carlo Di Mario was the first to apply high-frequency (40 MHz) IVUS for the study of human vascular lesions *in vitro* and compared it to histologic cross-sections (15). Compared with a rotating system, the phased array design offered significant advantages. It requires a smaller access sheath and is safer for use in tortuous arteries, as a mechanically rotating catheter generated friction against the arterial wall, which could hindered the generation of clear image (16, 17).

In the late 1990s and early 2000s, newer generations of IVUS systems were developed to address the limitations of earlier devices. A significant improvement was the reduction in the size of IVUS catheters, permitting easier access to smaller and more tortuous vessels (18). During this period, higher frequency transducers ranging from 40 to 60 MHz were introduced, to enhance lateral resolution (19). In 1998, (Endosonics, Rancho Cordova, CA) made a notable contribution by producing the only Food and Drug Administration (FDA)-approved system that combined balloon-transducer system that permitted stent delivery with immediate IVUS imaging post-depolyment (20). Furthermore, Yock et al. developed an innovative prototype that combined IVUS with atherectomy device, allowing imaging of regions requiring removal in real-time (21).

Beyond traditional grayscale IVUS, the late 2000s saw the utilization of backscattered IVUS radiofrequency data for tissue characterization, leading to the introduction of Virtual Histology-IVUS in 2002 (VH-IVUS) (12). This catheter-based technology uses IVUS-generated signals to create a color-coded map based on reflected signals from the artery wall (12, 22). In following years, advancements in IVUS system continued by different companies. For instance, in 2014 FDA cleared the Polaris imaging system by (Boston Scientific, Marlborough, MA) combines IVUS with fractional flow reserve (FFR) to additionally assess coronary flow. Furthermore, the integration of IVUS catheters with near-infrared spectroscopy (NIRS) has been explored, primarily for research purposes. This combination can quantify the lipid burden within coronary plaques.

### Optical coherent tomography

The concept of OCT originated in the late 1980s. Adolf Fercher presented the first two-dimensional *in vivo* depiction of a human eye fundus along a horizontal meridian based on white light interferometric depth scans was presented at the ICO-15 SAT conference 1990 (23). This technology was then pioneered by two independent research groups: one in Japan led by Naohiro Tanno, a professor at Yamagata University, and another in the USA led by David Huang, a professor at the Massachusetts Institute of Technology (MIT). Both groups studied OCT and patented their discoveries almost simultaneously (24, 25). In 1996, a collaborative effort between Brezinski et al. at Massachusetts General Hospital and Dr. Fujimoto and Dr. Tearney at MIT led to the development of one of the first OCT catheter prototypes (26). In the same year, James Fujimoto and Mark Brezinski conducted tests on their prototypes using segments of the human abdominal aorta postmortem, and for the first time identified atherosclerotic, calcified, and thin-walled lipid-filled plaques using their catheter system (27). However, a primary limitation of their early model was that observations were made primarily in an “in air” environment, without the presence of blood in the vessel wall. This limitation would be significant in the initial models of OCT systems.

Initial experiments correlating a limited number of excised coronary and aortic specimens with histology have demonstrated OCT's capability to resolve the microstructural features of atherosclerotic plaques (24). Furthermore, *in vivo* animal studies have shown the effectiveness of intravascular OCT catheters in directly imaging normal rabbit aortas and swine coronary arteries (28, 29). In a significant advancement in 2002, Guillermo Tearney presented the first results of intracoronary OCT in living patients (30). He used a prototype developed at Mass General hospital, and the OCT catheter was a non-commercial 1.06 mm (3.2 Fr) modified IVUS catheter. He compared OCT images with those generated by the traditional IVUS systems of the same arteries and commented on the significantly higher resolution that allowed for identification of fine details such as intimal hyperplasia, the boundaries between the internal and elastic lamina, thin fibrous caps, and other anatomical variations that could not be detected by IVUS. Nine months later, Yabushita et al. (27) presented the first study aimed at establishing OCT criteria for characterizing atherosclerotic plaques *in vitro*. This was done by correlating time domain (TD) -OCT images with histological findings.

In 1996, the ZEISS company (Jena, Germany) introduced the first OCT system (OCT 1) specifically designed for eye care (28). This initial commercial OCT system was based on the time-domain technology (29). Meanwhile, the inventors of OCT—Mark Brezinski MD, PhD, James Fujimoto PhD, and Eric Swanson MS—in collaboration with Carl Zeiss America, co-founded LightLab Imaging in 1997. LightLab Imaging has played a crucial role in the advancement of OCT technology. LightLab Imaging (Westford, MA) developed the first commercially available OCT imaging system, known as the M2/M3 TD-OCT Imaging System, along with the associated ImageWire catheter. The first generation of OCT systems was based on the TD modality, where tissue depth was determined by physically altering the distance to a reference mirror. However, this initial generation faced limitations due to the necessity of a bloodless field for imaging the arterial wall and its relatively low frame rate and pullback speed, which resulted in lower quality images (30).

In response to these challenges, newer generations of OCT systems have been developed to address many of the structural and technical limitations of first-generation systems. The concept of frequency-domain (FD) OCT, often referred to as spectral-domain OCT, was first proposed by Adolf Fercher et al. in 1995 (31). This FD system utilized a fixed mirror with a variable-wavelength light source and measured data as a function of time and wavelength (32). The first *in vivo* application of this technology in animals was by Seok Yun in 2006, who used FD-OCT to image the microstructure of long segments of the coronary arteries in swine (33). Tearney et al. were pioneers in testing the FD system within the coronary vasculature at the Wellman Center for Photomedicine, Massachusetts General Hospital, in 2008 (34). Subsequently, (LightLab Imaging, Westford, MA) responded to the limitations of the TD modality of the M2/M3 system by introducing a FD OCT system (C7-XR) with a Dragonfly imaging catheter. This advanced system was approved by the FDA and received CE marking in 2010. See Table 2 for a technical comparison between the M3 and C7-XR systems.

**Table 2:** Comparison between time domain optical coherence tomography (TD-OCT) system and frequency domain-OCT (FD-Oct).

Specifications	M3 (TD-OCT)	C7-XR (FD-OCT)
Axial resolution, μm	15–20	15-Dec
Lateral resolution, μm	39	19
Frame rate, fps	20	100
Lines/frame	240	500
Pullback speed, mm/sec	0.5–2.0	25-Oct
Scan diameter (FOV), mm	6.8	10
Tissue penetration, mm	2-Jan	2-Jan
Balloon occlusion	Highly recommended	Optional

(Lightlab Imaging, Westford, MA) was sold to Goodman Co. Ltd. in 2002. On May 19, 2010, this company was acquired by (St. Jude Medical, St Paul, MN) marking a significant milestone in the development and dissemination of OCT technology. They continue to manufacture the C7XR system, along with the IllUMIEN Optis system. The IllUMIEN Optis system has advanced to simplify OCT image processing, aiding in real-time decisions regarding stent diameter and length selection. In 2011, (St. Jude Medical, St Paul, MN) received approval for its ILUMIEN system, which innovatively combines FFR and OCT technologies.

### Development of a hybrid IVUS–OCT catheter

Recently, hybrid IVUS-OCT systems were developed to merge the advantages of both modalities into a single Catheter. In 2011, Yin et al. reported a modified miniaturized probe (OCT-US) that combined OCT and IVUS (35). Following this, in 2012, Li et al. introduced another hybrid system (33). Both prototypes were tested in healthy aortas of rabbits. However, these early models have limitations that preclude their use in clinical practice. The primary issues were the large size of the catheter, inability to accurately co-register OCT and IVUS images, and increased noise in the IVUS images due to electromagnetic interference from the motor.

A significant breakthrough occurred in 2018, when Sheth et al. (34) reported the first clinical use of a hybrid IVUS-OCT catheter. This iteration showcased beautifully co-registered images and met clinically acceptable specifications regarding size, speed, and resolution. The Novasight Hybrid™ system (Conavi Medical, Toronto, Canada), represents a significant advancement in this field. It was first tested in humans in 2018, and is now clinically available in the United States and Canada (34, 35). Another notable system is the dual-sensor system (TERUMO, Tokyo, Japan), which is incorporated in the AltaView and FastView systems. AltaView has received approval from the Pharmaceuticals and Medical Devices Agency (PMDA) in Japan, whereas FastView has been approved by both the PMDA and received the CE mark. Given the unique strengths and limitations of both IVUS and OCT, these hybrid catheters are theoretically poised to offer a more comprehensive evaluation of coronary artery diseases by leveraging the advantages of both modalities. As such, it raises the question of whether these novel devices will be able to outperform traditional IVUS and OCT systems in clinical practice.

### Near-infrared spectroscopy

Near-infrared spectroscopy (NIRS) represents a developing imaging modality that utilizes infrared light to characterize the lipid content of vulnerable plaques (36, 37). Observations of changes in the absorption of light have been recognized as early as 1876 (38) leading to a milestone NIRS paper by Professor Frans F. Jöbsis of Duke University in 1977 (39), who is regarded as the pioneer of medical applications of NIRS. First FDA approval for a device using NIRS technology was in 1993 for cerebral oximetry device for non-invasive detection of tissues oxygen saturation (40). However, Its vascular current utility is derived from its ability to identify high-risk lesions that may be prone to rupture and cause ensuant acute coronary syndromes (41). It was first tested on arterial systems in 1993 by Cassis and Lodder (42). It was first shown that NIRS could accurately characterize low-density lipoprotein cholesterol accumulation in hypercholesterolemic rabbit aortas. In 1996, NIRS was then used in humans to image lipid content in carotid plaques exposed at the time of surgery (43).

The early reports indicated that NIRS could be useful for characterizing plaque have been presented by Werner Jaross in 1999, who compared cholesterol content determined by NIR spectroscopy vs. that determined by reversed-phase, high-pressure liquid chromatography in human aorta specimens (44). Later in 2002, other group led by Pedro Moreno at the University of Kentucky later reported the NIRS ability to identify plaque composition in human aortic atherosclerotic plaques (45). They also reported the ability of NIRS to detect an inflamed thin-cap fibroatheroma. Pedro Moreno extended the study from aortic to coronary tissues and demonstrated the ability of NIRS to differentiate normal human coronaries tissues from diseased atherosclerotic plaques (46).

In August 2005, the new ultrafast NIRS system was tested in six patients at the Lahey Clinic by Dr. Sergio Waxman and Dr. Nesto who confirmed safety of the device (41). In 2008, Garcia-Garcia et al. (47) stated that detection of coronary vulnerable plaques *in vivo* is essential for studying their natural history and assessing potential treatment modalities and, therefore, may have an important impact on the prevention of acute myocardial infarction and death. At the same year on 2008, FDA gave approval for the first catheter-based LipiScan(TM) Coronary Imaging System developed by (InfraReDx, Burlington, MA). In 2009, Waxman et al. (48) reported their initial results from the SPECTACL (SPECTroscopic Assessment of Coronary Lipid) trial, a first-in-human multi-center, designed to demonstrate the applicability of the lipid core-containing plaques detection algorithm in living patients. They compared catheter based NIRS signals obtained from coronaries of patients vs. to those from autopsy specimens. The initial results confirmed feasibility and safety of the intravascular NIRS system. However, intracoronary NIRS imaging system received regulatory approval in USA (FDA approval), Europe (CE marked) and Japan (PMDA) in 2010, 2011 and 2014, respectively. In 2011, (InfraReDx, Burlington, MA) announcement the launch of its TVC Imaging System™, an enhanced version of the LipiScan™ IVUS Coronary Imaging System.

NIRS has been extensively validated for lipid-rich plaque detection against the gold-standard of histology and is the only FDA-approved method to identify coronary lipids. Several, studies have demonstrated the positive association between lipid burden in culprit lesion detected by NIRS and coronary events (49–51). In 2019, the Lipid-Rich Plaque (LRP) trial conducted by Ron Waksman demonstrated that NIRS imaging of non-obstructive territories in patients undergoing cardiac catheterization can help in identifying patients and segments at higher risk for subsequent non-culprit major adverse cardiovascular events (36). Based on the results of LRP trial, FDA announced the approval of NIRS for detection of high-risk plaques and patients in 2019. Following the LRP trial, PROSPECT II trial (52) by David Erling in 2021 reported similar results.

Following the clinical success of NIRS and its integration with IVUS, efforts have expanded to combine NIRS with other high-resolution modalities such as OCT. This trend toward multimodal imaging has led to the development of new hybrid systems. While some of these technologies remain at a very early stage and are not yet approved or commercially available, recent progress has led to the development of clinically usable systems. A notable example is the HyperVue Imaging System (SpectraWAVE, Inc), which received FDA clearance and integrates next-generation optical coherence tomography (DeepOCT) with near-infrared spectroscopy (NIRS) into a single catheter. This system offers enhanced depth penetration and resolution, enabling simultaneous plaque morphological assessment and lipid detection, co-registered with the coronary angiogram. In 2024, its first-in-human use demonstrated the feasibility of high-fidelity intracoronary imaging using this combined approach with artificial intelligence–assisted interpretation (53).

The availability of co-registered OCT–NIRS imaging represents a new frontier in coronary imaging, merging compositional and structural information in a single acquisition. This innovation may offer unprecedented precision in plaque characterization, improve PCI guidance, and ultimately support new paradigms in preventive and interventional cardiology.

## Conclusion

Since its inception, intracoronary imaging techniques, such as IVUS, OCT, and NIRS, have revolutionized cardiovascular medicine. We must acknowledge the groundbreaking work of the pioneers and inventors who made this possible. These high-resolution imaging tools provide detailed plaque characterization, significantly impacting percutaneous coronary intervention (PCI) optimization, and propelling our understanding of vascular biology. Although not yet fully adopted in routine clinical practice, the potential of intracoronary imaging remains vast. The integration of artificial intelligence, innovative technologies, and next-generation hardware and software promises to unlock ideal PCI outcomes, a deeper understanding of vascular biology, and, ultimately, improved prevention and treatment strategies for coronary artery disease. In addition, several hybrid technologies—such as fluorescence lifetime imaging-OCT (FLIm-OCT), intravascular photoacoustic-IVUS (IVPA-IVUS), fluorescence lifetime imaging-IVUS (FLIm-IVUS), near-infrared fluorescence-OCT (NIRF-OCT), and near-infrared fluorescence-IVUS (NIRF-IVUS) are currently under development, further underscoring the continuous innovation and evolution in the field of intravascular imaging.
